# Supplementary material for: P-TRAP: a Panicle Trait Phenotyping tool
Source: BMC Plant Biol. 2013 Aug 29;13:122. doi: 10.1186/1471-2229-13-122 (PMC3848748; doi:10.1186/1471-2229-13-122)
Supplement: Additional file 7 — P-TRAP source code. The Netbeans project that contains the source code of the application. [file 1471-2229-13-122-S7.zip › javadoc.zip!/javadoc/Index.html]

Javadoc for P-TRAP packages


# Javadoc for P-TRAP

| Name | Size | Last Modified | |
| --- | --- | --- | --- |
| org-basmati-workspace |  | 6/6/13 | 6:28:28 PM |
| org-bioalg-graphprocessor |  | 6/6/13 | 6:28:06 PM |
| org-bioalg-imageprocessor |  | 6/6/13 | 6:28:33 PM |
| org-bioalg-mathprocessor |  | 6/6/13 | 6:23:18 PM |
| org-bioalg-reportprocessor |  | 6/6/13 | 6:28:33 PM |
| org-bioalg-riceprojecttype |  | 6/6/13 | 6:28:33 PM |
| org-bioalg-skeletonprocessor |  | 6/6/13 | 6:28:33 PM |
| org-bioalg-widgetfactory |  | 6/6/13 | 6:28:33 PM |
| org-biolag-particleprocessor |  | 6/6/13 | 6:28:33 PM |
| org-netbeans-modules-riceoptions |  | 6/6/13 | 5:35:22 PM |
